# Malignant peritoneal mesothelioma as a rare cause of ascites: a case report

**DOI:** 10.1186/1752-1947-2-121

**Published:** 2008-04-25

**Authors:** Iftikhar Ahmed, Anastasios Koulaouzidis, Javaid Iqbal, Wong C Tan

**Affiliations:** 1 Department of Gastroenterology, North Cheshire Hospitals NHS Trust, Warrington, UK; 2 Department of Gastroenterology, Llandudno General Hospital, North Wales, UK; 3 Department of Medicine, District General Hospital NHS Trust UK

## Abstract

**Introduction:**

Peritoneal mesothelioma is a rare tumor with diagnostic and therapeutic problems. The peritoneum is the second most common site for development of mesothelioma, which in 30–45% of cases is associated with a synchronous pleural mesothelioma. Clinical symptoms and findings may be confusing and diagnosis can be easily overlooked especially in cases where there is no previous asbestos exposure.

**Case presentation:**

We report a case of malignant peritoneal mesothelioma in a 75-year-old woman who presented with ascites which, in the absence of inhalational exposure to asbestos, caused diagnostic confusion, and evaded radiological detection.

**Conclusion:**

We concluded from this case that Peritoneal Mesothelioma although rare but should be considered among the differential diagnosis of Ascites.

## Introduction

Malignant mesothelioma of the peritoneum is rare but rapidly fatal malignancy. The median survival rang from 5 to 12 months, mainly because of lack of effective treatment. The incidence is approximately one per 1,000,000; approximately one fifth to one third of all mesotheliomas are peritoneal. Half of reported cases have a history of asbestos exposure. The diagnosis of peritoneal mesothelioma is often delayed, in part because of the usually long latent period (peaking at 40–45 years from the time of initial exposure to asbestos) and because the common presenting symptoms of weight loss, usually with a full abdomen, malaise, and abdominal discomfort, are mild and nonspecific. Because of its unusual nature, the disease has not been clearly defined in terms of its natural history, diagnosis, or management. Treatment options with intravenous chemotherapy are far from satisfactory. However, because malignant peritoneal mesothelioma usually remains confined to the peritoneal cavity for most of its natural history, regional chemotherapy is an attractive option.

## Case presentation

A 75-year-old woman was admitted with a five-week history of progressive dyspnoea, ascites, leg edema and lethargy. She had a previous history of pulmonary tuberculosis 50 years earlier and hypertension which was well controlled with atenolol. She had never smoked and had no known previous exposure to asbestos. She had a family history of lung and large bowel cancer but no history of mesothelioma.

**Figure 1:**
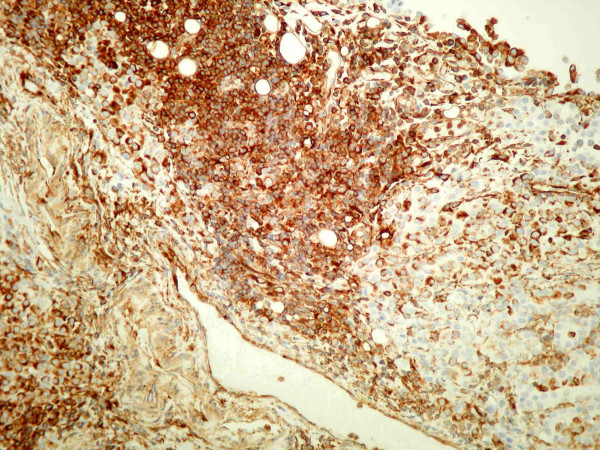
Sheaths of cells with fairly uniform nuclei containing prominent nucleoli consistent with poorly differentiated malignancy.

Clinical examination revealed bilateral pedal pitting edema but no signs of chronic liver disease. She had moderate ascites but normal cardiovascular and respiratory system examinations. Blood investigations including full blood count (FBC), urea and electrolytes, liver function tests (LFTs), inflammatory markers, hepatitis, viral and autoimmune screens were all normal. Initial ultrasound scan and computed tomography (CT) scan of the abdomen and pelvis were unable to demonstrate any significant abdominal pathology other than ascites. A diagnostic ascitic tap yielded transudate with a serum ascites-albumin gradient (SA-AG) of 32. Ascitic fluid culture and culture for acid-fast bacilli (AFB) were also negative. The ascites recurred despite multiple drainages. She had a repeat CT scan which showed extensive peritoneal nodularity and omental cake along with massive ascites. A diagnostic laparoscopy with omental biopsy was performed. It showed infiltration of the omentum with a poorly differentiated malignant tumor consisting of sheaths of cells with fairly uniform nuclei containing prominent nucleoli (Figure 1).

A barium enema and flexible sigmoidoscopy showed no bowel pathology apart from mild diverticular disease with pedunculated polyps which were found to be benign on histology. Immunochemical staining of the biopsy showed features consistent with diffuse mesothelioma. She was referred to the oncology department after a multidisciplinary meeting discussion and chemotherapy was planned with cisplatin and pemetrexed. She responded well initially and the ascites resolved with improved general wellbeing, but the treatment was later stopped when she developed side effects in the form of recurrent vomiting and general malaise. Currently she is having palliative treatment and is under regular oncology follow up.

## Discussion

Mesothelioma is a neoplasm originating from the mesothelial surface lining cells of serous cavities. Most often, mesotheliomas involve the serosal membranes of the pleura and peritoneum. Sometimes mesothelial proliferations are identified in other locations. On rare occasions, it has been reported in the tunica vaginalis of the testis [1,2]. Among all cases of mesothelioma, about 80% are pleural in origin. A causal relationship between asbestos exposure and pleural, peritoneal and pericardial malignant mesotheliomas has been suggested, the risk of cancer being correlated with cumulative exposure. There is some evidence that simian virus 40 may have some role in co-carcinogenesis [1,3].

Symptomatology is insidious and poses difficult problems in diagnosis and treatment. People with peritoneal mesothelioma generally present with one of two types of symptoms and signs: those with abdominal pain, usually localized and related to a dominant tumor mass with little or no ascites, and those without abdominal pain, but with ascites and abdominal distention [4].

Ultrasonography and CT scan of the abdomen can provide important information during the diagnostic process. Nevertheless, a definite diagnosis can only be established by laparoscopy or open surgery with biopsy to obtain histological examination along with immunocytochemical procedures. Laparoscopy is an important tool in the diagnosis of some unusual causes of ascites, such as primary mesothelioma, which are usually overlooked by other diagnostic modalities, such as ultrasound, CT and cytology of the ascitic fluid. However, laparoscopy can greatly complicate management by facilitating tumor dissemination to port sites [5].

Pathologically, a positive immunostain for calretinin has markedly increased the accuracy of diagnosis [1]. Prognosis, as determined by clinical presentation, the completeness of cytoreduction and gender, given that women survive longer than men with this condition, appears to be improved by the use of intraperitoneal chemotherapy.

Over the past decade, the management of these patients has evolved similarly to ovarian cancer treatment and now involves cytoreductive surgery, heated intraoperative intraperitoneal chemotherapy (HIIC) with cisplatin and doxorubicin, and early postoperative intraperitoneal paclitaxel. These perioperative treatments are followed by adjuvant intraperitoneal paclitaxel and second-look cytoreduction. This multimodality treatment approach with cytoreductive surgery and intraperitoneal chemotherapy has resulted in a median survival of 50 to 60 months [1,4].

## Conclusion

This case denotes the importance of considering the differential diagnosis of common clinical problems. Peritoneal mesothelioma, although uncommon, should be considered in people presenting with ascites, in particular in those where the initial diagnosis is not clear. A history of asbestos exposure may not be present and radiological investigations may miss the diagnosis so thorough clinical assessment and a broad-thinking approach is important.

## Abbreviations

AFB: acid-fast bacilli; CT: computed tomography; FBC: full blood count; HIIC: heated intraoperative intraperitoneal chemotherapy; LFT: liver function test; SA-AG: serum ascites-albumen gradient.

## Competing interests

The authors declare that they have no competing interests.

## Authors' contributions

IA was involved in writing the case, the literature review and obtaining patient consent. WCT was involved in proof reading and final refinement. AK was involved in proof reading and final refinement. JI was involved in the final revision and in making modifications following suggestions by the reviewers.

## Consent

Written informed consent was obtained from the patient for publication of this case report and accompanying images. A copy of the written consent is available for review by the Editor-in-Chief of this journal.

**Figure 2:**
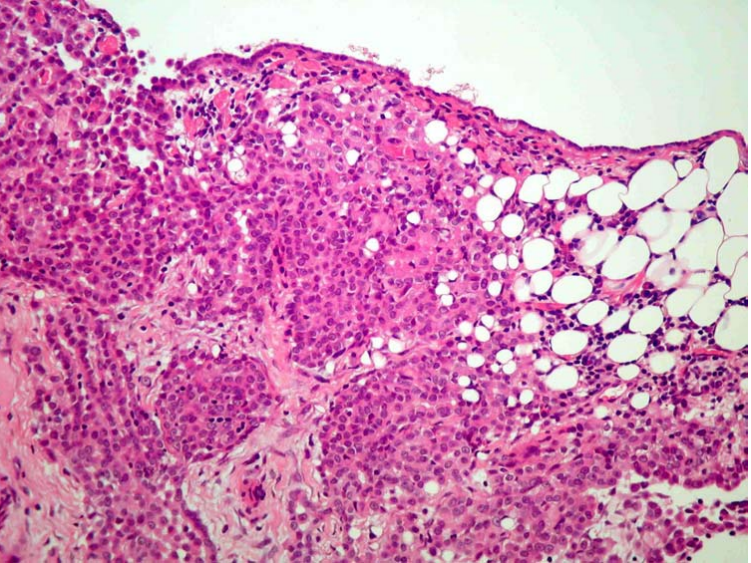
Sheaths of cells with fairly uniform nuclei containing prominent nucleoli consistent with poorly differentiated malignancy.

**Figure 3:**
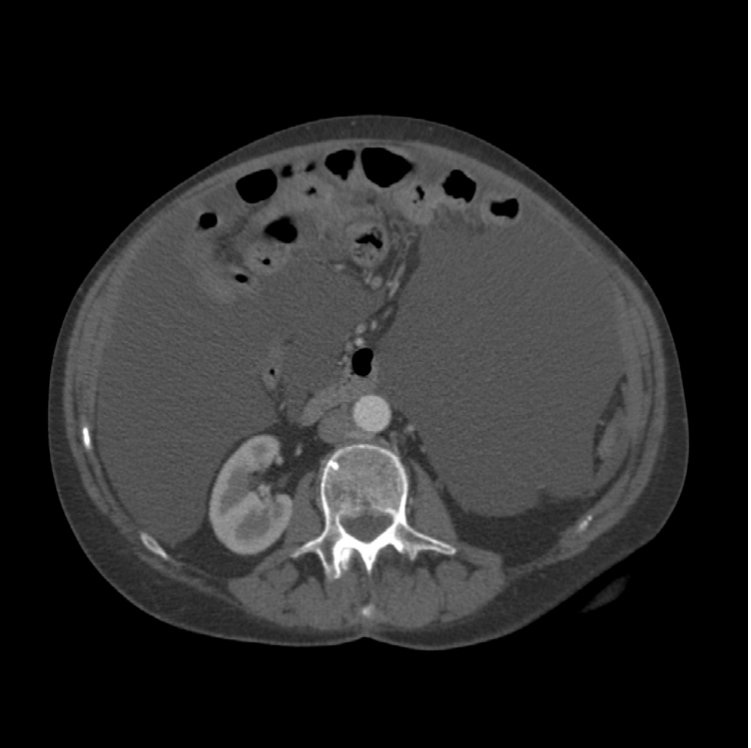
CT scan of the abdomen showing ascites.

**Figure 4:**
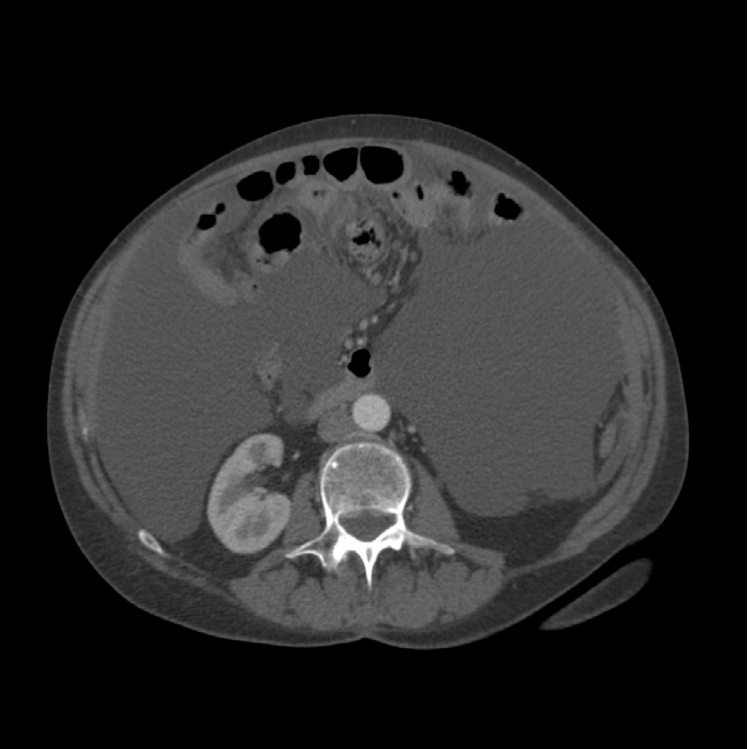
CT scan of the abdomen showing omental nodularity.
